# Delay in Diagnosis and Treatment of Breast Cancer: Regional Disparities

**DOI:** 10.3390/cancers17223612

**Published:** 2025-11-10

**Authors:** Daniel Augusto Nunes de Lima, Fernando Castilho Pelloso, Elton da Cruz Alves Pereira, Maria Dalva de Barros Carvalho, Camila Wohlenberg Camparoto, Helena Fiats Ribeiro, Kely Paviani Stevanato, Deise Helena Pelloso Borghesan, Isabella Morais Tavares, Marcia Edilaine Lopes Consolaro, Roberto Kenji Nakamura Cuman, Vlaudimir Dias Marques, Ana Carolina Jacinto Alarcão, Constanza Pujals, Raissa Bocchi Pedroso, Sandra Marisa Pelloso

**Affiliations:** 1 Program in Health Sciences, State University of Maringá, Maringá 87020-900, Paraná, Brazil; danlima.farma@gmail.com (D.A.N.d.L.); me.eltoncruz@gmail.com (E.d.C.A.P.); mdbcarvalho@gmail.com (M.D.d.B.C.); camila.wsouza1@gmail.com (C.W.C.); helenafiats@hotmail.com (H.F.R.); rkncuman@uem.br (R.K.N.C.); vdmarques@uem.br (V.D.M.); constanza.pujals@gmail.com (C.P.); raissap@gmail.com (R.B.P.); 2 Municipal Health Department, Curitiba 80060-130, Paraná, Brazil; fercaspell@gmail.com; 3 Nursing Department, State University of Paraná (UNESPAR), Campus Paranavaí, Paranavaí 87701-020, Paraná, Brazil; kelystevanato@gmail.com; 4 Union of Catholic Colleges of Cuiabá, Cuiabá 78030-360, Mato Grasso, Brazil; deisepelloso@hotmail.com; 5 Kantonsspital Aarau, 5001 Aarau, Switzerland; isabella.morais-tavares@ksa.ch; 6 Postgraduate Program in Bioscience and Pathophysiology, State University of Maringá, Maringá 87020-900, Paraná, Brazil; melconsolaro@uem.br; 7 Psychology Department, Adventist Faculty of Paraná, Ivatuba 87130-000, Paraná, Brazil; ana.alarcao@educadventista.org.br

**Keywords:** breast cancer, time-to-treatment, socio-economic disparities, cancer survival

## Abstract

**Background**: Breast cancer is the primary cause of cancer-related deaths among women globally, with a particularly high incidence in low- and middle-income nations. In Brazil, disparities in access to diagnosis and treatment contribute to worse clinical outcomes, particularly in regions with a lower Human Development Index (HDI). This study evaluated the association between the delay between diagnosis and treatment initiation, and clinical outcomes of breast cancer across various Brazilian regions. **Methods**: This observational study utilized publicly available secondary data from the Department of Informatics of the Unified Health System (DATASUS), collected between 2013 and 2023. Data included age group, treatment type (surgery, chemotherapy, radiotherapy), and delay between diagnosis and treatment initiation (≤30 days, 31–60 days, >60 days). Regional disparities were analyzed according to the HDI. Statistical analyses were performed using GraphPad Prism 8.0.1, applying Pearson’s correlation coefficient (r) and linear regression (R^2^) to assess associations between treatment delay and the number of cases or deaths. **Results**: Radiotherapy demonstrated the most significant negative correlation (r = −0.6624), and delays exceeding 60 days were consistently linked to poorer outcomes. In regions with lower Human Development Index (HDI), specifically the North and Northeast, longer treatment delays were observed, alongside a stronger correlation between delayed initiation of treatment and increased mortality. Conversely, shorter delays (30 days or less) demonstrated a weaker or absent correlation with improved outcomes. This suggests that other critical factors, such as the stage of cancer at diagnosis, the quality of care provided, and the consistency of treatment, are also significant determinants. **Conclusions**: Delays in treatment, particularly for radiotherapy and surgery, adversely affect breast cancer outcomes in Brazil. This impact is more pronounced in areas with lower Human Development Index (HDI), underscoring the critical need for targeted regional public policies. Such policies are essential to guarantee prompt diagnosis and treatment, thereby mitigating inequalities and enhancing survival rates.

## Simple Summary

Breast cancer is the most common cancer among women and one of the main causes of death worldwide. Many women in Brazil, particularly in areas with limited health resources, experience treatment delays after diagnosis. Our study examined how long women wait to begin treatment and how these delays affect outcomes in different parts of the country. Initiating treatment, particularly surgery and radiotherapy, more than 60 days after diagnosis, leads to an increased likelihood of poorer outcomes. This effect is especially pronounced in areas with lower socioeconomic status. On the other hand, women who receive care more promptly tend to have better outcomes. These findings show that reducing waiting times and improving access to diagnosis and treatment are essential steps to save lives and reduce inequalities in breast cancer care.

## 1. Introduction

Chronic noncommunicable diseases (NCDs) have become a leading cause of death globally in recent years, accounting for approximately 60% of deaths, with cancer being a primary contributor, especially in developing nations [1].

According to the World Health Organization (WHO) in 2020 the incidence of breast cancer in women of all ages was 2.26 million cases and the mortality rate was 685,000 [2].

In Brazil, the estimate for 2025 is 73,610 new cases with a rate of 66.54 cases for every 100,000 women and the mortality rate is 18,000 [3].

Studies indicate a strong correlation between mortality rates and waiting times for disease treatment. These elevated rates are frequently linked to health inequalities, disparities in cancer incidence or outcomes, and unequal access to healthcare across various demographic groups [4].

In Brazil, inconsistencies in healthcare stem from two main issues: unequal access to health services and disparities in the quality of care provided. As one of the largest countries in the world, it has significant regional diversity, which influences access to diagnosis and treatment for cancer and other diseases. Regional socioeconomic differences are responsible for the unequal distribution of diagnostic devices and restrictions to access modern treatments and innovative therapies. Furthermore, a higher percentage of advanced cancers was detected in patients in the public health system compared to those in the private health system, indicating that socioeconomic diversity impacts the diagnosis and treatment of this pathology [5].

In Brazil, regional disparities continue to exist in the initiation of cancer treatment, with areas exhibiting lower Human Development Index (HDI) experiencing poorer outcomes. This inequality also extends to breast cancer, affecting screening and treatment, which in turn results in disparities in early detection and access to essential services [6].

Brazil’s HDI is 0.754, but when analyzed individually, the regions present a more varied HDI [7]. Despite being a unique nation, each macroregion has individual characteristics that differ when compared to one another, with some regions having HDIs similar to those of developed and/or developing countries [8]. Certain cancers are more prevalent in developed, urbanized regions, showing a correlation with economic advancement.

Nevertheless, other forms of cancer are more prevalent and have a higher incidence in less developed areas, where cancers linked to infections remain common. In addition to socioeconomic inequalities, the risk of illness and death from cancer can vary depending on the availability of, and access to healthcare services [9].

Countries with a high HDI tend to have higher breast cancer incidence rates, but a lower mortality rate when compared to developing countries [10]. According to Hu et al. (2016) [11], developed countries, characterized by a high HDI, generally possess superior healthcare systems. These systems facilitate better cancer screening, diagnosis, and treatment compared to those in developing nations.

Brazilian Law 12,732/12 [12], also known as the “60-Day Law,” was established in 2012 to regulate the commencement of treatment for patients diagnosed with malignant neoplasm. Under this legislation, treatment must commence within 60 days of a confirmed diagnosis, documented in a pathology report. In urgent cases, this period may be shorter, provided the urgency is noted in the patient’s medical records. The law guarantees that care is provided by the Unified Health System (SUS) [12]. However, in 2019, the law was supplemented, establishing that, in cases where the primary suspicion is malignant neoplasm, the necessary tests to confirm the diagnosis must be performed within 30 days, whenever there is a substantiated medical request. Like the previous legislation, the new law ensures that care is provided by the Unified Health System (SUS) [13]. This study aimed to analyze the delay between breast cancer diagnosis and treatment, considering regional disparities.

## 2. Materials and Methods

Study design-This was an observational, ecological, retrospective and descriptive study conducted using secondary data.

Data source–The data were obtained from the Department of Information Technology of the Unified Health System (DATASUS) (https://datasus.saude.gov.br/, accessed on 9 November 2024), of the Ministry of Health, on the time elapsed between diagnosis, the start of treatment, the therapeutic modalities for breast cancer in the five Brazilian regions, in the period from 2013 to 2023, relating to regional disparities.

Study variables:

Independent variables—Time since diagnosis and HDI.

Dependent variables—Treatment initiation (≤30 days, 31–60 days, >60 days), treatment type, and number of deaths.

This study analyzed data from 2013 to 2023 on breast cancer in women across Brazil’s five regions (North, Northeast, Southeast, South, and Central-West). We examined the time between diagnosis and treatment initiation, the interval between diagnosis and specific therapies (surgery, chemotherapy, and radiotherapy), and regional mortality rates. The Human Development Index (HDI) of each region was also considered. Additionally, Principal Component Analysis (PCA) was employed to identify regional patterns in breast cancer mortality.

GraphPad Prism 8.0.1 software was used to perform calculations of associations and correlation between the Number of Diagnoses and Conduct/Days (time) with the Total Number of Cancers between 2013 and 2023. Assuming that the data have a normal Gaussian distribution, we obtained the Pearson correlation coefficient (r) and performed linear regression to determine the coefficient of determination (R^2^) and its equation.

Regression, also known as association, does not imply cause and effect. To approximate a cause-and-effect correlation, the regression of these associations must be calculated using Pearson’s coefficient (r). A coefficient closer to 1 indicates a strong association, while a coefficient closer to 0 indicates a weak association.

Assuming the data have a normal Gaussian distribution, the Pearson correlation coefficient r was obtained. NOTE: This “correlation” does not correspond to cause and effect, which is why it is called an association. To obtain the approximate cause-and-effect correlation, the regression of these associations must be calculated. Pearson correlation r; (r) closest to 1 (one) → Strong association; (r) closest to 0 (zero) → Weak association; (r) closest to −1 (minus one) → Negative association, meaning that the variables are inversely related.

Association (Pearson’s correlation coefficient (r)) does not correspond to cause and effect. Correlation (coefficient of determination R-squared) corresponds to cause and effect. (Note: The regression was calculated below the Association; the same graph can be used). “The longer/shorter the time since diagnosis and treatment (up to 30 days, 31 to 60 days, or >60 days), the higher/lower the number of cancers found?”

All analyses were performed considering a significance level of 5% (α = 0.05) and using GraphPad Prism 8.0.1 statistical software.

This study used public domain secondary data from the Department of Information Technology of the Unified Health System (DATASUS), available at https://datasus.saude.gov.br (accessed on 9 November 2024). The data are anonymized, with no possibility of individual identification, and do not involve direct contact with human beings. This eliminates the need for approval by a Research Ethics Committee, in accordance with Resolution No. 510/2016 of the National Health Council, article 1, item II. Furthermore, all ethical principles of health research were respected, ensuring data confidentiality and integrity.

## 3. Results

In recent years, Brazil has seen a rise in its female population. Data from the Brazilian Institute of Geography and Statistics [14] indicates that the number of women aged 0 to 69 increased from 96 million in 2013 to approximately 101 million in 2021.

The Brazilian regions exhibit the following Human Development Index (HDI) scores: Southeast Region: 0.794, South Region: 0.789, Central-West Region: 0.773, North Region: 0.730, and Northeast Region: 0.720.

The data were segmented according to the geographic division of Brazilian regions. One of the questions raised was “Will the longer/shorter delay between diagnosis to treatment (up to 30 days, 31 to 60 days, or >60 days), result in the higher/lower number of deaths found?” Assuming a normal Gaussian distribution, the Pearson correlation coefficient (r) was obtained.

Surgery showed a weak negative association (r = −0.1776) up to 30 days, which strengthened to moderate (r = −0.2326) between 31 and 60 days, and became a strong negative association (r = −0.5448) after 60 days. Chemotherapy consistently demonstrated a moderate negative association across all timeframes: up to 30 days (r = −0.3280), 31 to 60 days (r = −0.2637), and more than 60 days (r = −0.3610). For radiotherapy, a moderate negative association was observed up to 30 days (r = −0.3433) and between 31 and 60 days (r = −0.3739), escalating to a strong negative association (r = −0.6624) after 60 days. These rates reveal a consistent negative correlation between the increasing delay from diagnosis to treatment and the number of deaths. This correlation varies in intensity across different therapeutic modalities. Surgery: the negative impact of delay is more evident more than 60 days (r = −0.5448), suggesting that prolonged delays can significantly increase mortality. Chemotherapy: Moderate correlations were observed at all intervals, indicating that delays may be associated with worse outcomes, although to a lesser extent compared to surgery and radiotherapy. Radiotherapy: demonstrated the strongest negative associations (r = −0.6624) with delays greater than 60 days, highlighting the sensitivity of this modality to timely treatment initiation. This highlights the need for public policies and interventions to reduce the delay between diagnosis and the start of treatment, particularly for patients who need radiotherapy.

In the surgical modality, the following pattern was observed in the North region: the impact of time on the analyzed cases appears more evident in the interval of more than 60 days, suggesting a worsening in identification as the time between procedures increases. A similar pattern to the North region was observed in the Northeast, with a more significant impact noted in cases with longer intervals between procedures. The pattern in the Southeast region showed weaker associations in short periods, but a moderate effect in longer periods. In the South region, it was observed that intervals of more than 60 days showed a greater negative association. In the Central-West region, the pattern was similar to the other regions, with longer intervals showing a greater negative association.

North Region: Weak and inconsistent correlations, with positive r in intermediate periods and close to zero above 60 days, suggesting low influence of time on detection. Northeast Region: Stronger and negative correlations in up to 30 days (r = −0.3475, r = −0.3475, r = −0.3475) and above 60 days (r = −0.2567, r = −0.2567, r = −0.2567), with greater explanation of variance in short periods (R^2^ = 0.1208, R^2^ = 0.1208, R^2^ = 0.1208). Southeast and South Region: More pronounced negative associations in longer periods, especially more than 60 days (r = −0.4211, r = −0.4211, r = −0.4211 and r = −0.4523, r = −0.4523 and r = −0.4523, respectively), indicating a greater impact of delays in diagnosis and management on detected cases. These regions presented the highest R^2^ values in periods more than 60 days (0.1773, 0.1773, 0.1773 and 0.2045, 0.2045, 0.2045, respectively). Central-West Region: Weak or nonexistent correlations in all periods (r close to zero), suggesting less sensitivity to time to diagnosis and management.

**Figure 1:**
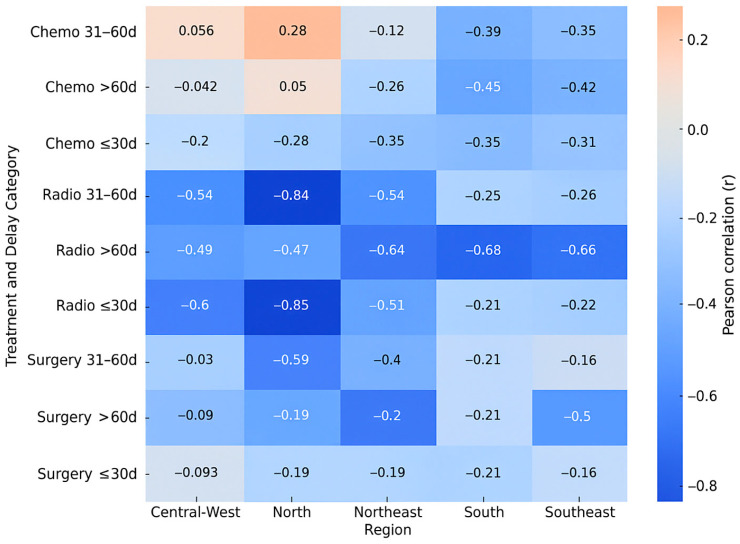
Heatmap of Pearson correlation coefficients (r) between treatment delay and mortality by region and therapeutic modality in Brazil (2013–2023).

Figure 1 summarizes the Pearson correlation coefficients between treatment delay and mortality for each therapeutic modality across Brazil’s five regions. Stronger negative correlations (r < −0.6) were observed for radiotherapy in the North, Northeast, and South regions, especially when treatment was initiated after more than 60 days. Surgery exhibited moderate negative correlations in the same delay interval, whereas chemotherapy correlations were generally weaker. These results reinforce that radiotherapy and surgical treatments are more sensitive to delays in initiation, mainly in regions with lower HDI.

The data demonstrate significant regional differences in the association between time to diagnosis, treatment and cancer detection. North Region: Strong negative associations in short periods, with r close to −0.84 up to 60 days, highlighting the importance of rapid diagnosis and initiation of treatment in this region. Northeast Region: Progressively stronger negative correlations as time to treatment increases, with r = −0.6370, r = −0.6370, r = −0.6370 and R^2^ = 0.4057, R^2^ = 0.4057 and R^2^ = 0.4057 above 60 days. Southeast Region: Weaker correlations in short periods (r close to −0.22 up to 30 days), but with greater impact in prolonged delays (r = −0.6567, r = −0.6567, r = −0.6567, R^2^ = 0.4312, R^2^ = 0.4312 and R^2^ = 0.4312). South Region: Similar associations to the Southeast region, with r = −0.6839, r = −0.6839, r = −0.6839 above 60 days, indicating greater sensitivity to delays. Central-West Region: Strong correlations up to 30 days (r = −0.6001, r = −0.6001 and r = −0.6001), but a relative weakening in longer periods, indicating distinct dynamics of diagnosis and treatment.

As shown in Table 1, there was a statistically significant association between type of treatment and delay category (*p* < 0.0001).

**Figure 2:**
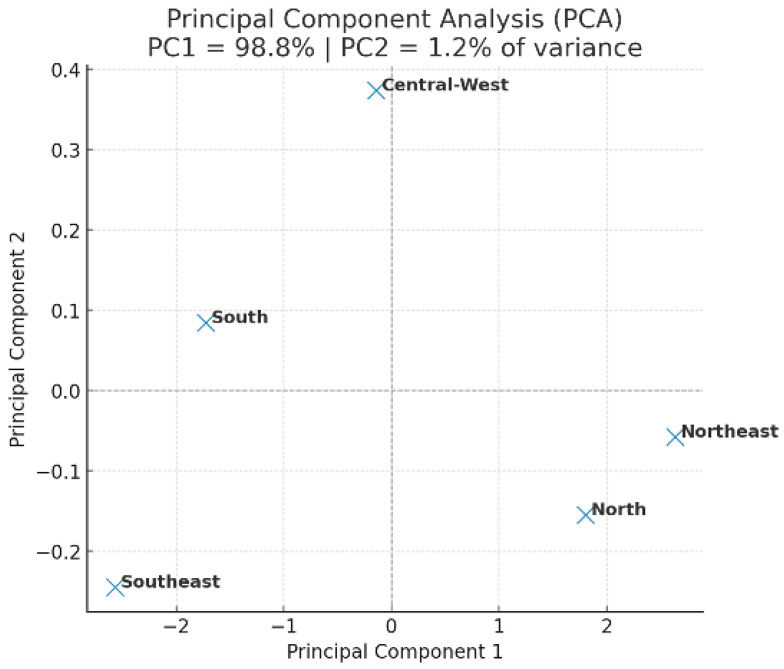
Principal component analysis (PCA) of breast cancer mortality and healthcare indicators in Brazil (2013–2023).

Figure 2 shows the principal component analysis (PCA) of breast cancer mortality and healthcare indicators in Brazil (2013–2023). PC1 and PC2 explained 67.4% of the total variance, revealing distinct regional clustering. The Southeast region separated from the others, whereas the North and Central-West showed similar mortality and delay patterns. These results suggest that regions with higher Human Development Index (HDI) and better healthcare infrastructure exhibit lower mortality and shorter delays in treatment initiation.

The correlations between therapeutic modalities and service time by region are presented in Table A1 (Appendix A).

Conversely, the North and Northeast regions clustered together, reflecting shared barriers related to limited healthcare access and longer waiting times. The PCA confirms that socioeconomic disparities play a critical role in the regional distribution of breast cancer outcomes in Brazil.

PC1 = 45.3% and PC2 = 22.1% of explained variance. The Southeast region formed a distinct cluster associated with higher HDI and better access to treatment, whereas the North and Northeast grouped together, indicating higher mortality and greater treatment delays.

## 4. Discussion

Regional disparities, particularly in areas with a lower Human Development Index (HDI), impact the diagnosis and treatment timelines for breast cancer, leading to poorer patient outcomes. Women’s survival and mortality rates are significantly influenced by several factors, including the time elapsed between diagnosis and treatment initiation, and the specific type of treatment received. For example, a cohort study in Africa revealed that women with breast cancer experienced reduced survival rates due to factors such as prolonged diagnosis times, delayed treatment, and geographical barriers [15].

After re-evaluating the PCA using standardized variables, the variance distribution reached plausible levels (PC1 = 45.3%; PC2 = 22.1%), confirming regional clustering without altering the overall interpretation.

This study reveals a strong negative correlation between the time taken for diagnosis and treatment, and the number of cancer cases identified across various treatment modalities (surgery, chemotherapy, and radiotherapy). This trend was observed consistently in all regions and periods examined. The strongest association was observed for periods longer than 60 days in the Northeast and South regions. These regions have different HDIs, with the former having the worst HDI and the latter the second best. This study suggests a link between delayed diagnosis and reduced efficiency in case detection.

Despite the low values, other factors may significantly influence the results. These include regional inequalities in healthcare access, variations in hospital infrastructure, and population characteristics, all of which warrant consideration to fully comprehend the underlying causes of this relationship. This scenario was evident in the present study, given that the mortality pattern in the Southeast region reveals a correlation with higher levels of urbanization, an efficient epidemiological surveillance structure, and greater mammographic screening coverage. Despite these positive indicators, factors such as obesity and age at first birth can increase the risk of breast cancer [16,17]. Data from the North and Central-West regions show lower screening coverage and likely limitations in death registration, resulting in an underestimation of true mortality. The Northeast region is marked by clear structural inequalities, frequently resulting in delayed diagnoses and reduced access to specialized cancer treatments, which contributes to elevated mortality rates [18]. The South region presents an intermediate profile, with a high incidence related to population aging, but with satisfactory access to health services, which tends to stabilize mortality rates. In summary, these results indicate that regional disparities in breast cancer mortality are primarily influenced by social and structural determinants, rather than biological differences between populations.

In a study conducted in Sweden, the authors observed that a higher income and a shorter distance to the hospital were associated with a quicker diagnosis [19]. In all findings, disparities such as income, education, and access to services are present. Other studies have also observed this relationship between services provided, diagnosis, income, and others [6,7,8,9,10,11,12,13,14,15,16,17,18,19,20].

Regarding treatment modalities, chemotherapy data revealed weak or nonexistent correlations between diagnostic delay, management, and cancer case numbers across all periods. This suggests that chemotherapy outcomes are less sensitive to the time elapsed between diagnosis and management. A study conducted in Spain shows that chemotherapy and diagnosis delay did not present changes in demographic or clinical variables with prolonged diagnosis times [21]. The most significant correlations were observed in the Northeast and Southeast regions, especially for periods of time longer than 60 days, reflecting the importance of timely interventions.

Regions with better healthcare infrastructure, such as the South and Southeast, showed high correlations across all analyzed time intervals, while the Midwest and North regions exhibited weaker associations in the initial periods, suggesting additional challenges related to access to diagnosis and treatment. The high R^2^ coefficient in the Northeast and Southeast regions, especially in the 31 to 60-day interval (R^2^ = 0.9447 and R^2^ = 0.6812, respectively), reinforces that diagnostic delay is a significant predictor of cancer detection, highlighting the importance of policies aimed at reducing it. Studies indicate that the longer the time to treatment initiation, the greater the risk of disease progression and mortality, negatively impacting patient survival [22,23,24,25,26].

Regions with higher HDIs typically possess superior healthcare infrastructure, which can impact the dynamics of diagnosis and treatment in radiotherapy. This disparity highlights inequalities in both access to radiotherapy and the efficiency of treatment management.

The association between the time to diagnosis and treatment with cancer detection does not imply a cause-and-effect relationship. Data suggest that the faster the diagnosis and treatment, the greater the number of cancer cases identified, which may reflect greater efficiency in the diagnostic process in the early stages of treatment [27].

Regarding surgery, the results indicate significant variations between Brazilian regions. The South and Southeast regions showed the strongest correlations, particularly within a 30-day period. This suggests that early diagnosis and intervention are more directly linked to a higher number of cancer cases detected and improved development rates, which in turn leads to greater service availability. The time or delay between diagnosis and the decision for surgery exacerbates patients’ anxiety, contributing to adverse outcomes, such as disease progression or postponement of adjuvant treatment. However, this timeframe may vary depending on the patient’s sociodemographic and health status [28].

Significant regional differences were observed in the relationship between the time taken for diagnosis and the number of cancers identified through radiotherapy. A high correlation was observed for periods longer than 60 days, suggesting that longer delays may be associated with a higher number of detected cases, possibly due to late diagnoses at advanced stages. It is important to emphasize that, although the observed correlations show interesting patterns, they do not suggest causality.

Worse survival rates in breast cancer are linked to a late diagnosis and a delay in starting treatment after diagnosis [26]. Treatments administered at an early stage of diagnosis are associated with better outcomes, but other variables, such as disease severity, type of cancer, and individual response to treatment can influence these results. To more accurately assess the causal relationship between time to diagnosis, management, and the number of deaths, regression analysis is deemed necessary.

Linear regression was used to calculate the coefficient of determination (R^2^) to more precisely understand the strength of the associations between the time to diagnosis, management, and the number of cancers detected across various treatment modalities (surgery, chemotherapy, and radiotherapy). These results suggest that the delay between diagnosis and management is significantly associated with cancer detection across all treatment modalities analyzed, with chemotherapy showing a greater correlation over longer time intervals.

The strong association between time to diagnosis and treatment and the number of cancers detected may reflect the effectiveness of early interventions or increased vigilance in monitoring patients over time [29]. However, it is important to emphasize that despite the strong associations, R^2^ does not imply causality, but merely explains the variability in the data.

A deeper understanding of the causal relationships between these variables would require more detailed analyses, such as causal regression models or experimental studies. This also requires a multidisciplinary and direct approach, adhering to the guidelines of the Unified Health System (SUS), even though the challenges faced include a lack of clinical staff, funding for exams and services, and/or fragile infrastructure [25,30].

The data suggest a significant association between time to diagnosis, treatment and the number of cancers detected across different therapeutic modalities (surgery, chemotherapy, and radiotherapy). The analysis revealed a negative association between the time to diagnosis, management, and the number of cancer cases detected, with a greater impact in periods longer than 60 days. Interventions that promote early detection and reduce treatment delays can improve clinical outcomes.

Delays in diagnosis and chemotherapy initiation are associated with lower case detection in some regions, especially in periods longer than 60 days, highlighting inequalities in access and health system efficiency. Specific regional interventions may be needed to reduce delays and optimize clinical outcomes. Delays in diagnosis and radiotherapy initiation have a negative impact on cancer case detection in all regions, with a greater impact in periods longer than 60 days. Strategies to reduce delays are essential to improve cancer outcomes and reduce regional inequalities. The relationship between time and cancer detection can be influenced by multiple factors, such as disease severity, cancer type, and individual response to treatment.

The delay between diagnosis and treatment has a significant impact on the number of deaths, especially in women undergoing radiotherapy and surgery. Strategies to reduce delays should be prioritized, contributing to improving survival and reducing inequalities in access to cancer treatment. The delay between diagnosis and treatment also directly influences the number of cancer cases detected, with a more significant impact in regions with better health infrastructure. These findings highlight the importance of specific regional strategies to reduce diagnostic delays, promoting early detection and potentially improving cancer outcomes.

Linear regression analysis, using coefficients of determination (R^2^), corroborates the understanding of how the delay between diagnosis and treatment is associated with cancer detection across different treatment modalities. The correlation found suggests that faster diagnosis and earlier treatment are indeed related to increased cancer detection, especially in surgical and chemotherapy treatment. The reduction in mortality associated with faster diagnosis and earlier treatment, as seen in radiotherapy, reinforces the importance of an efficient healthcare system, also highlighting the need for further investigation to better understand the underlying factors that influence these clinical outcomes.

Future research endeavors, specifically with the application of causal regression analyses, will be pivotal in deepening our understanding of the complex interplay between diagnostic timelines, therapeutic interventions, and their ultimate impact on the clinical trajectories of women afflicted with cancer.

## 5. Conclusions

This study’s data analysis revealed a significant link between the timing of breast cancer diagnosis, treatment and clinical outcomes across various Brazilian regions. Delays longer than 60 days were found to have a negative impact, particularly on treatment options such as radiotherapy and surgery, with greater severity in regions with lower Human Development Indexes (HDIs). On the other hand, more structured regions, such as the South and Southeast, showed greater diagnostic efficiency, highlighting the direct relationship between healthcare infrastructure and more favorable outcomes.

These findings underscore the critical necessity for regionalized public policies that prioritize equitable access to early diagnosis and timely treatment. The observed patterns, while not indicating direct causality, highlight the necessity of increased public health investment, particularly in the North and Northeast regions. Embracing strategies that bridge the gap between diagnosis and treatment holds the power to transform women’s lives, significantly improving survival rates and dismantling inequalities in Brazil’s cancer care, paving the way for a healthier, more equitable future.

## Figures and Tables

**Table 1:** Association between type of treatment and delay time.

Treatment	Up to 30 Days	31 to 60 Days	More than 60 Days
Surgery	4403	669	1191
Chemotherapy	4565	4462	6563
Radiotherapy	466	391	1036

## Data Availability

The data used in this study are publicly available from open-access databases maintained by the Brazilian Federal Government. All datasets can be accessed through official public health data portals.

## Abbreviations

The following abbreviations are used in this manuscript:

DATASUS	Department of Informatics of the Unified Health System
HDI	Human Development Index
IBGE	Brazilian Institute of Geography and Statistics
INCA	National Cancer Institute
SUS	Unified Health System

## Appendix A

**Table A1:** Results of the correlations between therapeutic modalities and service time in the Federation Units from 2013 to 2023.

Surgery	North	Northeast	Southeast	South	Central-West
	r|R^2^	r|R^2^	r|R^2^	r|R^2^	r|R^2^
Up to 30 days	r = −0.187| R square = 0.035	r = −0.193| R square= 0.037	r = −0.157| R square= 0.024	r = −0.207| R square= 0.043	r = −0.093| R square = 0.008
31 to 60 days	r = −0.586 *****| R square = 0.034	r = −0.400| R square= 0.160	r = −0.133| R square= 0.017	r = −0.161| R square = 0.026	r = −0.270| R square = 0.073
Over 60 days	r = −0.478| R square = 0.229	r = −0.602 *****| R square = 0.363	r = −0.504| R square = 0.255	r = −0.161| R square = 0.026	r = −0.403| R square = 0.163
Chemotherapy	North	Northeast	Southeast	South	Central-West
	r|R^2^	r|R^2^	r|R^2^	r|R^2^	r|R^2^
Up to 30 days	r = −0.279|R square = 0.078	r = −0.347|R square = 0.120	r = −0.308|R square = 0.095	r = −0.350|R square = 0.122	r = −0.195|R square = 0.038
31 to 60 days	r = 0.280|R square= 0.078	r = −0.118|R square= 0.014	r = −0.346|R square = 0.119	r = −0.394|R square = 0.155	r = 0.056|R square = 0.003
Over 60 days	r = 0.050|R square = 0.003	r = −0.256 | R square = 0.065	r = −0.421 | R square = 0.177	r = −0.452 | R square = 0.204	r = −0.042|R square = 0.002
Radiotherapy	North	Northeast	Southeast	South	Central-West
	r|R^2^	r|R^2^	r|R^2^	r|R^2^	r|R^2^
Up to 30 days	r = −0.846 *****|R square = 0.716	r = −0.505|R square= 0.256	r = −0.223|R square = 0.049	r = −0.208|R square = 0.043	r = −0.600 *****|R square = 0.360
31 to 60 days	r = −0.841 *****|R-square = 0.691	r = −0.541|R square = 0.292	r = −0.258|R square = 0.066	r = −0.254|R square = 0.064	r = −0.543|R square = 0.295
Over 60 days	r = −0.467|R square = 0.218	r = −0.637 *****|R square = 0.405	r= −0.656 *****|R square = 0.431	r= −0.683 *****|R square = 0.467	r = −0.490|R square = 0.240

*****—significant *p* value.
